# Identification of biomarkers in ductal carcinoma *in situ *of the breast with microinvasion

**DOI:** 10.1186/1471-2407-8-287

**Published:** 2008-10-06

**Authors:** Yasuhiro Okumura, Yutaka Yamamoto, Zhenhuan Zhang, Tatsuya Toyama, Teru Kawasoe, Mutsuko Ibusuki, Yumi Honda, Ken-ichi Iyama, Hiroko Yamashita, Hirotaka Iwase

**Affiliations:** 1 Department of Breast and Endocrine Surgery, Graduate School of Medical and Pharmaceutical Sciences, Kumamoto University, Honjo 1-1-1, Kumamoto 860-8556, Japan; 2 Department of Oncology and Immunology, Graduate School of Medical Sciences, Nagoya City University, Kawasumi 1, Mizuho-ku, Nagoya 467-8601, Japan; 3 Department of Pathology, Kumamoto University Hospital, Honjo 1-1-1, Kumamoto 860-8556, Japan

## Abstract

**Background:**

Widespread use of mammography in breast cancer screening has led to the identification of increasing numbers of patients with ductal carcinoma *in situ *(DCIS). DCIS of the breast with an area of focal invasion 1 mm or less in diameter is defined as DCIS with microinvasion, DCIS-Mi. Identification of biological differences between DCIS and DCIS-Mi may aid in understanding of the nature and causes of the progression of DCIS to invasiveness.

**Methods:**

In this study, using resected breast cancer tissues, we compared pure DCIS (52 cases) and DCIS-Mi (28 cases) with regard to pathological findings of intraductal lesions, biological factors, apoptosis-related protein expression, and proliferative capacity through the use of immunohistochemistry and the TdT-mediated dUTP-biotin nick end labeling (TUNEL) method.

**Results:**

There were no differences in biological factors between DCIS and DCIS-Mi, with respect to levels of estrogen receptor, progesterone receptor, and human epidermal growth factor receptor type 2. The frequency of necrosis and positive expression ratio of survivin and Bax were significantly higher in DCIS-Mi than in DCIS. In addition, apoptotic index, Ki-67 index, and positive Bcl-2 immunolabeling tended to be higher in DCIS-Mi than in DCIS. Multivariate analysis revealed that the presence of necrosis and positive survivin expression were independent factors associated with invasion.

**Conclusion:**

Compared with DCIS, DCIS-Mi is characterized by a slightly elevated cell proliferation capacity and enhanced apoptosis within the intraductal lesion, both of which are thought to promote the formation of cell necrotic foci. Furthermore, the differential expression of survivin may serve in deciding the response to therapy and may have some prognostic significance.

## Background

Ductal carcinoma *in situ *(DCIS) is thought to be a precursor of invasive ductal carcinoma (IDC) and is defined as a lesion in which cancer cells do not grow beyond the basal membrane of the mammary duct [1]. Since the introduction of mammography in breast cancer screening, increasing numbers of DCIS are now being identified [2]. About 10 years ago, DCIS accounted for only 1–5% of all newly diagnosed cases of breast cancer, whereas the frequency has increased recently to 15–20% [3,4]. According to the criteria of the American Joint Committee on Cancer (AJCC), IDC with a microscopic focus of invasion less than or equal to 0.1 cm in the longest dimension, is defined as T1mic [5]. In situations where there are multiple foci of microinvasion, the classification is based on the largest focus, rather than the sum of all the individual foci and therefore, if the size of the largest focus is less than 0.1 cm, the lesion will still be defined as T1mic [5]. This ductal carcinoma *in situ *with microinvasion, known as DCIS-Mi, is identified in 10–20% of cases thought to be DCIS, through the use of preoperative diagnostic imaging and cytology; it is considered to represent the interim stage in the progression from DCIS to IDC [6-8]. Histopathological factors including architectural subtypes, nuclear grade, the presence of necrosis, tumor size, and the distance to the surgical stump have already been reported as predictors of the risk of local recurrence in DCIS [9-11]. Further analyses of the biological parameters will make the prediction of local recurrence in DCIS more accurate. At present, various biological parameters have been reported as prognostic factors for DCIS. Overexpression of human epidermal growth factor receptor 2 (HER2), decreased expression of estrogen receptor (ER), overexpression of p53, and positive Ki-67 staining, have all been shown to correlate with nuclear grade in both DCIS and IDC [8,12-16]. It has also been reported that endometase/matrilysin-2 and tissue inhibitors of metalloproteinase-2 and -4 are overexpressed in the invasive component of DCIS [17]. However, there have been few reports investigating the status of apoptosis and apoptosis-related factors in DCIS. In particular, the *survivin *gene, shown to be expressed in proliferating cells such as fetal and various cancer cells, is known to be a member of the inhibitor of apoptosis protein (IAP) family, which promotes cell proliferation and inhibits apoptosis [18-21].

Tumor progression may be induced or associated by alterations in the proliferative capacity and the apoptosis potential, and the findings may find some basic and applied interpretations especially useful for confirming a diagnosis, and in building up prognostic criteria. Therefore, apoptosis-related factors (apoptotic index, survivin, Bcl-2, Bax), cell proliferation/cycle-related factors (Ki-67, p53, p21, cyclinD1, Rb), and other factors are analyzed in DCIS and DCIS-Mi in this investigation.

## Methods

### Patients

The study population consisted of 52 patients with DCIS and 28 with DCIS-Mi, diagnosed by postoperative histopathological examinations after undergoing surgery for the diagnosis of non-invasive or invasive ductal carcinoma of the breast at the Kumamoto University Hospital or the Nagoya City University Hospital from 1990 to 2005. The median age was 56 years old (range, 36–86) in patients with DCIS and 50 (range, 32–71) in patients with DCIS-Mi. The median follow-up period was 62 months (range, 13–140) in DCIS and 54 (range, 14–182) in DCIS-Mi (Table 1). To date, of the DCIS-Mi patients, one has had a recurrence in the contralateral breast and another has developed bone metastasis. There have been no deaths. The information was obtained from the patient records. The ethics committee of Kumamoto University and Nagoya City University approved the study protocol and confirmed that it was conducted in accordance with the guidelines of the 1975 Declaration of Helsinki. Informed consent was obtained from all patients before or after surgery. Histological diagnosis was made by 2 specialized pathologists. The histological subtypes were determined on the predominant area occupying most of the lesion, although in some cases multiple histological subtypes were mixed. When there were multiple foci of microinvasion, if the size of the largest focus was less than 0.1 cm, the lesion was regarded as DCIS-Mi.

Histological variables, including classification of histological subtypes, grade of nuclear atypia, and the presence of necrotic foci, were evaluated on slides stained with hematoxylin-eosin (HE). According to the Van Nuys classification [22], cases were classified into three groups: high-nuclear grade (Grade 3); non-high nuclear grade with necrosis (Grade 2); and non-high nuclear grade without necrosis (Grade 1). In some cases, microinvasion was confirmed by staining the basal membrane with type IV collagenase [23].

**Table 1:** Histological characteristics of intraductal component in ductal carcinoma *in situ *(DCIS) and in ductal carcinoma *in situ *with microinvasion (DCIS-Mi)

	**DCIS**	**DCIS-Mi**	
Number of patients	52	28	
Median follow-up period, months (range)	62 (3–140)	54 (14–182)	
Median age, years (range)	56 (36–86)	50 (32–71)	
Architectural classification			
comedo	8 (15.4%)	10(35.7%)	*P *= 0.073
Papillary	21 (40.4%)	10 (35.7%)	(comedo *vs *others)
cribriform	15 (28.8%)	4 (14.3%)	
solid	4 (7.7%)	4 (14.3%)	
low papillary	4 (7.7%)	0 (0)	
Nuclear Grade			
Grade 1	21 (40.4%)	7 (25)	*P *= 0.16
Grade 2	29 (55.8%)	18 (64.3)	(1 vs. 2,3)
Grade 3	2 (3.9%)	3 (10.7)	
Necrosis			
absent	25 (48.1%)	4 (14.3%)	*P *= 0.0017
Present	27 (51.9%)	24 (85.7%)	
Van Nuys Classification*			
Grade 1	25 (48.1%)	4 (14.3%)	*P *= 0.0017
Grade 2	25 (48.1%)	21 (75%)	(1 vs. 2,3)
Grade 3	2 (3.9%)	3 (10.7%)	

* Van Nuys Classification: Grade 1 = non-high nuclear grade without necrosis; Grade 2 = non-high nuclear grade with necrosis; Grade 3 = high-nuclear grade.

### Immunohistochemistry

Resected specimens were fixed in 10% buffered-formalin and embedded in paraffin within 24–48 hours. Four-μm sections were cut from this paraffin block and mounted on adhesive-coated slides (Aminosilane-coated slides; MATSUNAMI GLASS Co. Ltd., Japan) for immunohistochemistry [15]. The sections were deparaffinized in xylene (5 × 5 minutes) and ethanol (2 × 100%, 1 × 90%, 1 × 80%, 1 × 70%). Subsequently, antigen retrieval was performed in citrate buffer at pH 9 for survivin as previously described [24], using a microwave oven (after boiling, 3 × 10 minutes at 750W or 4 × 15 minutes at 170W). After leaving at room temperature for 20 minutes, sections were immersed in phosphate-buffered saline (PBS). Endogenous peroxidase was blocked by incubation with 3% H_2_O_2 _in methanol for 10 minutes. Sections were washed with PBS (3 × 5 minutes) followed by blocking with 5% skimmed milk for 20 minutes [25].

Immunohistochemical staining was performed by the avidin-biotin complex (ABC) method using the Vectastain ABC kit for ER, progesterone receptor (PgR), HER2, Ki-67, survivin, p53, p21, cyclin D1, and Rb, (monoclonal antibodies, DAKO, Netherlands) and by the immuno-enzyme polymer method (indirect method, EnVision system, DAKO, Netherlands) using Histofine Simple Stain MAX PO (Nichirei Bioscience, Tokyo Japan) for Bcl-2 and Bax [26]. Sections were incubated with dilutions of primary antibodies for 60 minutes at room temperature. After washing with PBS (3 × 5 minutes), sections were incubated with biotinylated secondary antibodies for 30 minutes. Following this, the sections were washed again with PBS (3 × 5 minutes) and then reacted with an ABC solution for 30 minutes at room temperature. After a further washing with PBS (3 × 5 minutes), the sections were stained with 3, 3'-diaminobenzidine (DAB) substrate solution.

In the immuno-enzyme polymer (indirect) method, sections were incubated with dilutions of primary antibodies at 4°C overnight. After washing with PBS (3 × 5 minutes), sections were reacted with the Histofine Simple Stain MAX PO (mouse or rabbit), washed again with PBS (3 × 5 minutes), and then stained with DAB solution [25]. Nuclear staining of all sections was done with hematoxylin. The sections were mounted after dehydration (1 × 70%, 1 × 80%, 1 × 90%, 2 × 100% ethanol) and penetration (3 × xylene for 3 minutes). Details of the primary antibodies used in this study are shown in Table 2.

**Table 2:** Details of immunohistochemistry and the antibodies

**Antibody**	**Source**	**Clone**	**Dilution**	**Pretreatment**	**Citrate buffer**	**Incubation**	**Detection system**
ER	DAKO	1D5	1/50	4 × 15 min MW	pH6	1 h RT	ABC
PgR	DAKO	636	1/800	4 × 15 min MW	pH6	1 h RT	ABC
HER2	DAKO		1/300	4 × 15 min MW	pH6	1 h RT	ABC
Ki-67	DAKO	MIB-1	1/50	3 × 10 min MW	pH9	1 h RT	ABC
Bcl-2	DAKO	124	1/50	3 × 10 min MW	pH9	ON 4°C	En-Vision
Bax	DAKO		1/50	3 × 10 min MW	pH6	ON 4°C	En-Vision
Survivin	SantaC	D-8	1/50	3 × 10 min MW	pH9	1 h RT	ABC
p53	DAKO	DO-7	1/50	3 × 10 min MW	pH9	1 h RT	ABC
p21	DAKO	SX118	1/25	3 × 10 min MW	pH9	1 h RT	ABC
cyclinD1	NovoC	P2D11F11	1/50	3 × 10 min MW	pH9	1 h RT	ABC
Rb	NovoC	13A10	1/100	3 × 10 min MW	pH6	1 h RT	ABC

ER = estrogen receptor; PgR = progesterone receptor; MW = microwave; RT = room temperature; ON = overnight; Santa C = Santa Cruz; NovoC = NovoCastra.

### Evaluation of immunohistochemistry

For Ki-67 expression, the percentage of cancer cells with positively-stained nuclei was determined. Nuclear labeling of more than 10% of all observed cancer cells was regarded as positive for ER, PgR, p53, p21, cyclin D1, and Rb [25]. HER2 expression was categorized into no staining (0), weak staining (+1), moderate staining (+2), or strong staining (+3), and was regarded as positive only when cancer cells showed strong staining (+3) [15]. Staining of more than 5% of all observed cancer cells was regarded as positive for survivin, Bcl-2, and Bax [20,27].

### Apoptosis

Apoptosis was detected by the TdT-mediated dUTP-biotin nick end labeling (TUNEL) method using ApopTag Peroxidase In Situ Apoptosis Detection Kit (S7100; Chemicon International, CA, USA) [28]. After deparaffinization, sections were incubated with Proteinase K for 15 minutes at room temperature and immersed in pure water. After this, the sections were incubated with 3% H_2_O_2 _for 5 minutes at room temperature followed by washing with PBS (2 × 5 minutes). Sections were incubated with equilibration buffer for at least 10 minutes at room temperature and then with working strength TdT enzyme at 37°C for 60 minutes, at which point the sections were incubated with working strength stop/wash buffer for 10 minutes after being agitated for 15 seconds at room temperature, followed by washing with PBS (2 × 1 minute). Sections were then incubated with anti-digoxigenin conjugate for 30 minutes at room temperature followed by washing with PBS (4 × 2 minutes). Sections were stained with DAB solution, dehydrated, and mounted. To quantitate apoptosis, the mean number of positive nuclei per 1000 counted nuclei/field was determined in three fields within the cancerous areas of the intraductal components. The mean number of positive nuclei was defined as the apoptotic index.

### Statistical methods

The statistical tests were utilized to examine the difference in each factor between DCIS and DCIS-Mi. Quantitative analyses (Ki-67 and apoptotic index) were performed using the Mann-Whitney U test; all other factors were analyzed with the Chi-squared test and Fisher's exact test. For all comparisons, differences were considered significant when the *P*-value was less than 0.05. Statistical analyses were performed using the statistical software JMP Japanese Version 4.0 (SAS).

## Results

### Histological evaluation

Results showed that the comedo type tended to be found more frequently in DCIS-Mi (36%) than in DCIS (18%), although this difference did not reach statistical significance (*P *= 0.073). There was no difference in nuclear grade between the two groups but a necrotic focus was found at a significantly higher frequency in DCIS-Mi (85.7%) than in DCIS (51.9%) (*P *= 0.0017). According to the Van Nuys classification, which evaluates both nuclear grade and necrosis, the grade of malignancy was significantly higher in DCIS-Mi (14.3%, Grade 1) than in DCIS (48.1%, Grade 1) (*P *= 0.0017, Grade 1 *vs*. Grade 2 and 3) (Table 2).

### Relative expression of hormone receptors, HER2, and Ki-67: DCIS vs. DCIS-Mi

**Figure 1:**
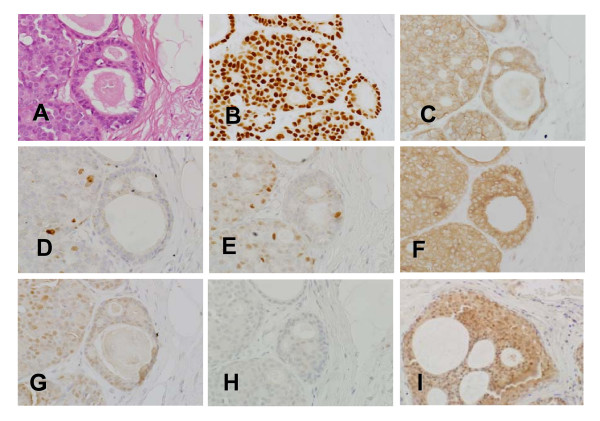
**Representative microscopic views of immunohistochemical detection.** Intraductal component found in patients with DCIS, using **A**. hematoxylin-eosin (HE) staining, the frames show cells stained for **B**. estrogen receptor (ER), **C**. HER2, **D**. Ki-67, **E**. p21, **F**. BCL-2, **G**. BAX, and **H**. Survivin 1, respectively.  **I**. Survivin 2 shows moderately positive Survivin staining detected in another patient with DCIS. (original magnification × 200)

There was no significant difference between DCIS and DCIS-Mi in expression levels of universal biological factors such as ER, PgR, and HER2. The Ki-67 index tended to be higher in DCIS-Mi (22.8 ± 2.0%) than in DCIS (17.9 ± 1.5%), although the difference was not statistically significant (*P *= 0.052) (Table 3). Representative microscopic views of immunohistochemical detection were shown in Fig. 1.

**Table 3:** Hormone Receptor status, HER2 status and Ki-67 index in DCIS and DCIS-Mi

	**DCIS**	**DCIS-Mi**	***P*-value**
Estrogen Receptor			
positive	38 (73.1%)	19 (67.9%)	0.62
negative	14 (26.9%)	9 (32.1%)	
Progesterone Receptor			
positive	37 (71.2%)	16 (57.1%)	0.21
Negative	15 (28.9%)	12 (42.9%)	
c-erbB-2			
0, 1+,2+	43 (82.7%)	22 (78.6%)	0.65
3+	9 (17.3%)	6 (21.4%)	
Ki-67 index			
positive rate %	17.9 ± 1.5	22.8 ± 2.0	0.052
mean ± SD			

DCIS = ductal carcinoma *in situ*; DCIS-Mi = ductal carcinoma *in situ *with microinvasion; SD = standard deviation.

### Relative expression of apoptosis-related markers: DCIS vs. DCIS-Mi

The relative expressions of the apoptosis-related markers in DCIS and DCIS-Mi are shown in Table 4. Apoptotic index tended to be higher in DCIS-Mi (0.29 ± 0.06%) than in DCIS (0.17 ± 0.04%) (*P *= 0.082). The expression of Bax, a promoting factor of apoptosis, was significantly higher in DCIS-Mi (96.4%) than in DCIS (71.2%) (*P *= 0.0028). The expression of survivin, an inhibiting factor of apoptosis, was also significantly higher in DCIS-Mi (85.7%) than in DCIS (55.8%) (*P *= 0.0048), while the expression of another apoptosis inhibitor (Bcl-2) tended to be found more frequently in DCIS-Mi (78.6%) than in DCIS (58.8%), although this did not reach statistical significance (*P *= 0.071). There was no difference in the expression levels of p53, p21, cyclin D1, and Rb between DCIS and DCIS-Mi (Table 4). Because no cases were detected showing nuclear staining of survivin, in this study cases were regarded positive only when cytoplasmic staining was detectable. Based on this limitation the survivin expression showed a positive correlation with Bax expression (*P *= 0.038) and cases positive for survivin tended to show a higher apoptotic index (*P *= 0.077) (Table 5).

**Table 4:** Apoptosis-related factors in DCIS and DCIS-Mi

	**DCIS**	**DCIS-Mi**	***P*-value**
Apoptotic Index (N/1000)	1.7 ± 0.4	2.9 ± 0.6	0.082

Bcl-2			
negative	21 (41.2%)	6 (21.4%)	0.071
Positive	30 (58.8%)	22 (78.6%)	
Bax			
negative	15 (28.8%)	1 (3.6%)	0.0028
Positive	37 (71.2%)	27 (96.4%)	
Survivin			
negative	23 (44.2%)	4 (14.3%)	0.0048
positive	29 (55.8%)	24 (85.7%)	
p53			
positive	39 (76.5%)	23 (82.1%)	0.55
Negative	12 (23.5%)	5 (17.9%)	
p21			
positive	36 (69.2%)	15 (53.6%)	0.17
negative	16 (30.8%)	13 (46.4%)	
cyclinD1			
positive	36 (70.6%)	18 (64.3%)	0.57
Negative	15 (29.4%)	10 (35.7%)	
Rb			
positive	35 (68.6%)	23 (82.1%)	0.18
negative	16 (31.4%)	5 (17.9%)	

DCIS = ductal carcinoma *in situ*; DCIS-Mi = ductal carcinoma *in situ *with microinvasion.

**Table 5:** Relationship among apoptosis-related factors

	**Apoptosis index**	**Survivin**	**Bax**
Bcl-2	0.11	0.65	0.042
Bax	0.018	0.038	-
Survivin	0.077	-	-

### Univariate and multivariate analyses for factors affecting invasion

Univariate analysis for factors affecting invasion beyond the mammary ducts identified the presence of necrosis, positive Bax expression, and positive survivin expression to be significant factors and revealed apoptotic index, positive Bcl-2 expression, and Ki-67 positivity to have a weak correlation with invasion. Multivariate analysis revealed that the presence of necrosis (*P *= 0.017, odds ratio 5.19, 95% Confidence Interval; CI 1.47–22.7), and positive survivin expression (*P *= 0.044, odds ratio 3.98 95%CI 1.11–17.2) were independent factors associated with invasion (Table 6).

**Table 6:** Univariate and multivariate analyses according to microinvasion

	Univariate analysis			Multivariate analysis		
	Odds ratio	95% CI	*P*-value	Odds ratio	95% CI	*P*-value

Necrosis (present ***vs***. absent)	5.56	1.84–20.91	0.0017	5.19	1.45–22.7	0.017
Apoptotic index (≧1000 of positive cells ***vs***. < 1/1000)	0.40	0.14–1.04	0.061	0.82	0.24–2.75	0.74
Ki-67 index (≧20% of positive cells ***vs***. < 20%)	0.44	0.15–1.18	0.10	0.89	0.24–3.24	0.86
Bcl-2 (positive ***vs***. negative)	2.57	0.93–7.95	0.071	3.49	1.04–13.3	0.051
Bax (positive ***vs***. negative)	10.92	2.02–203.9	0.0028	5.21	0.70–111.1	0.16
Survivin (positive ***vs***. negative)	4.76	1.57–17.9	0.0048	3.98	1.11–17.2	0.044

CI, confidence interval

## Discussion

The investigation of the biological factors of DCIS-Mi may yield some explanations to the progression from DCIS to IDC. In this study, we examined the intraductal components of both DCIS and DCIS-Mi for expression of these factors, but not the invasive components. The reasons for this were: the purpose of this study was to determine the difference between the intraductal components of DCIS and DCIS-Mi; it was difficult to evaluate invasive foci because of their small size; in some cases, invasive foci were undetectable on slides for immunohistochemical staining; in histological diagnosis, it was important to evaluate the most predominant area of each section. Results from the present analysis showed that the frequency of necrosis was significantly higher in DCIS-Mi compared with DCIS.

The important result obtained from our analysis of apoptosis-related factors was that the positive rate of survivin expression was significantly higher in DCIS-Mi than in DCIS (*P *= 0.0048). The *survivin *gene, shown to be expressed in proliferating cells such as fetal and various cancer cells, is known to be a member of the IAP family which promotes cell proliferation and inhibits apoptosis. In particular, because survivin is expressed specifically in cancer cells and proliferating cells, this gene is likely to be involved in cell proliferation and/or differentiation [29,30].

Previous reports have shown that, in invasive ductal carcinoma of the breast, survivin expression is significantly higher in the invasive foci than in the intraductal components and that high nuclear expression of survivin in the invasive foci is associated with a higher risk of recurrence and mortality [19,30]. However, there have also been reports that showed no correlation between survivin expression and prognosis in patients with breast cancer [27,31], suggesting that its prognostic value is controversial. With the antibody used in this present study, survivin-specific staining in the nucleus was barely detectable, and therefore we relied upon cytoplasmic staining for the evaluation of survivin expression. Reasons for the lack of nuclear expression in this study remain unclear and must be sought in further study on survivin splice variants.

There have been many studies reporting that Bcl-2, an apoptosis inhibitor, shows a positive correlation with hormone receptors in breast cancer tissues and is a favorable prognostic factor [26]. In addition, it has been reported that the expression of an apoptosis promoter Bax is associated with the degree of local recurrence in DCIS [32]. Furthermore, the relation between Bcl-2 and Bax is under debate with some reports showing a direct correlation between them and others showing no such correlation [33].

In our present results, the Bax expression was significantly higher (*P *= 0.0028) and the Bcl-2 expression tended to be slightly higher (*P *= 0.071) in DCIS-Mi compared with DCIS. These results show a positive correlation between Bax and Bcl-2 and their increased expression with the progression from DCIS to DCIS-Mi. Thus, this may suggest that the specific interaction between Bax and Bcl-2 is involved in apoptosis during this progression.

In our present study, we also found a tendency for higher apoptotic and Ki-67 indices in DCIS-Mi relative to DCIS. It is well known that these indices are higher in invasive ductal carcinoma of the breast than in fibroadenoma, a benign breast tumor. In DCIS, it has been shown that proliferation and apoptosis markers are higher in poorly-differentiated tumors than in well-differentiated tumors, which has led some investigators to believe that the difference between DCIS and invasive carcinoma is a relative decrease in apoptosis [26]. Taken together, it has been suggested that both cell proliferation and apoptosis are enhanced in DCIS-Mi as compared to DCIS.

Furthermore, multivariate analysis for apoptosis-related factors affecting invasion, identified the presence of necrosis (*P *= 0.017) and high survivin expression (*P *= 0.044) as statistically significant factors. We also demonstrated that the frequency of apoptosis (*P *= 0.077) and nuclear grade (*P *= 0.081) tended to be higher in cases with positive immunolabeling for survivin, suggesting that survivin could be a marker reflecting the biological characteristics of DCIS and DCIS-Mi. In addition, there was a positive correlation between the expression of the anti-apoptotic factor survivin and that of an apoptosis promoter Bax (*P *= 0.038). These findings suggest that a balance is maintained in DCIS-Mi between survivin and Bax.

These results revealed that, compared to DCIS, DCIS-Mi is histologically associated with necrotic focus more commonly, and with a significantly higher expression of apoptosis-related factors, including survivin and Bax. In other words, DCIS-Mi is characterized by a slightly elevated cell proliferation ability and a tendency for enhanced apoptosis, which could increase cell death and subsequently promote the formation of necrotic foci [34]. In clinical settings, survivin may prove to be a useful marker to indicate a difference in biological features between DCIS and DCIS-Mi, and it could become one of the parameters used to determine whether adjuvant systemic therapy or surviving-inhibiting therapy should be given to patients with DCIS.

## Conclusion

Compared with DCIS, DCIS-Mi is characterized by a slightly elevated cell proliferation capacity and enhanced apoptosis within the intraductal lesion, which are thought to promote the formation of cell necrotic foci. Furthermore, survivin is one of the markers indicating the difference in biological features between DCIS and DCIS-Mi, and further investigation of this marker is required to determine if this difference in expression could serve as an indicator for identifying which patients would benefit from adjuvant systemic therapy.

## Competing interests

The authors declare that they have no competing interests.

## Authors' contributions

HI conceived the design and drafted the manuscript. YO, YY, ZZ performed the immunohistochemistry and the statistical analysis. TT, MI, HY collected the patients' data. YH and KI participated in the histological study design. HI supported the study financially. All authors read and approved the final manuscript.

## Pre-publication history

The pre-publication history for this paper can be accessed here:


